# Supplementary material for: Evidence for sample selection effect and Hawthorne effect in behavioural HIV prevention trial among young women in a rural South African community
Source: BMJ Open. 2018 Jan 10;8(1):e019167. doi: 10.1136/bmjopen-2017-019167 (PMC5781067; doi:10.1136/bmjopen-2017-019167)
Supplement: Author's manuscript [file bmjopen-2017-019167.draft_revisions.pdf]

BMJ Open

BMJ Open is committed to open peer review. As part of this commitment we make the peer review history of every article we publish publicly available.

When an article is published we post the peer reviewers' comments and the authors' responses online. We also post the versions of the paper that were used during peer review. These are the versions that the peer review comments apply to.

The versions of the paper that follow are the versions that were submitted during the peer review process. They are not the versions of record or the final published versions. They should not be cited or distributed as the published version of this manuscript.

BMJ Open is an open access journal and the full, final, typeset and author-corrected version of record of the manuscript is available on our site with no access controls, subscription charges or pay-per-view fees (http://bmjopen.bmj.com).

If you have any questions on BMJ Open's open peer review process please email editorial.bmjopen@bmj.com

Evidence for sample selection effect and Hawthorne effect in behavioral HIV prevention trial among young women in a rural South African community

Journal:	*BMJ Open*
Manuscript ID	bmjopen-2017-019167
Article Type:	Research
Date Submitted by the Author:	15-Aug-2017
Complete List of Authors:	Rosenberg, Molly; Indiana University School of Public Health-Bloomington, Epidemiology and Biostatistics<br>Pettifor, Audrey; University of North Carolina at Chapel Hill, Department of Epidemiology, School of Public Health<br>Twine, Rhian; University of the Witwatersrand, MRC/Wits Rural Public Health and Health Transitions Research Unit (Agincourt), School of Public Health, Faculty of Health Sciences<br>Hughes, James; University of Washington School of Public Health, Biostatistics<br>Gómez-Olivé, F. Xavier; University of the Witwatersrand, Medical Research Council/Wits Rural Health and Health Transitions Unit (Agincourt), School of Public Health, Faculty of Health Sciences<br>Wagner, Ryan; University of the Witwatersrand, Johannesburg, MRC/Wits Rural Public Health & Health Transitions Research Unit (Agincourt)<br>Sulaimon, Afolabi; University of the Witwatersrand, 5. MRC/Wits Rural Public Health and Health Transitions Research Unit (Agincourt)<br>Tollman, Stephen; University of the Witwatersrand, School of Public Health, Faculty of Health Sciences<br>Selin, Amanda; University of North Carolina Chapel Hill, Carolina Population Center<br>MacPhail, Catherine; University of the Witwatersrand School of Clinical Medicine, Wits Reproductive Health and HIV Institute (WRHI); University of New England, School of Health<br>Kahn, Kathleen; University of the Witwatersrand, Medical Research Council/Wits Rural Health and Health Transitions Unit (Agincourt), School of Public Health, Faculty of Health Sciences
**Primary Subject Heading**:	Epidemiology
Secondary Subject Heading:	Global health, HIV/AIDS, Research methods
Keywords:	Hawthorne effect, selection effect, HIV prevention, adolescents, South Africa

SCHOLARONE™
Manuscripts

For peer review only

Title: Evidence for sample selection effect and Hawthorne effect in behavioral HIV prevention trial among young women in a rural South African community

Authors: Molly Rosenberg[1,2], Audrey Pettifor[3,4,5], Rhian Twine[5], James P. Hughes[6], F. Xavier Gomez-Olive[2,5,7], Ryan G. Wagner[5,10], Afolabi Sulaimon[5], Stephen Tollman[5,7,10], Amanda Selin[4], Catherine Macphail[5,8,9], Kathleen Kahn[5,7,10]

Author affiliations:

1. Department of Epidemiology and Biostatistics, Indiana University School of Public Health-Bloomington
2. Center for Population and Development Studies, Harvard University
3. Department of Epidemiology, University of North Carolina-Chapel Hill
4. Carolina Population Center, University of North Carolina-Chapel Hill
5. MRC/Wits Rural Public Health and Health Transitions Research Unit (Agincourt), School of Public Health, Faculty of Health Sciences, University of the Witwatersrand, Johannesburg, South Africa
6. Department of Biostatistics, University of Washington
7. INDEPTH Network, Accra, Ghana
8. School of Health and Society, University of Wollongong, New South Wales, Australia
9. Wits Reproductive Health and HIV Institute, University of the Witwatersrand, Johannesburg, South Africa
10. Umeå Centre for Global Health Research, Division of Epidemiology and Global Health, Department of Public Health and Clinical Medicine, Umeå University, Umeå, Sweden

Corresponding author:
Molly Rosenberg
1025 E. 7th Street, Office C032
Bloomington, IN 47405
Email: rosenmol@indiana.edu
Phone: +1 (812) 856-2509
Fax: +1 (812) 855-9107

Word count: 2999/3000

Author contributions: MR, AP, RT, and KK conceived the study. MR conducted the analysis and wrote the first draft of the manuscript. AP, RT, JPH, FXGO, RGW, AS, ST, AS, CM, and KK were involved in the design of the parent studies and/or in collection, storage, and analysis of data from the parent study. All authors contributed to the interpretation of the findings, critical review of the manuscript, and approval of the final manuscript as submitted.

Data sharing statement: Agincourt HDSS data access can be requested at the following link: http://www.agincourt.co.za/index.php/data/

For peer review only - http://bmjopen.bmj.com/site/about/guidelines.xhtml

Abstract (252/300 words)

Objectives: We examined the potential influence of both sample selection effects and Hawthorne effects in the behavioral HIV Prevention Trial Network (HPTN) study 068, designed to examine whether cash transfers conditional on school attendance reduce HIV acquisition in young South African women. We explored whether school enrollment among study participants differed from the underlying population, and whether differences existed at baseline (sample selection effect) or arose during study participation (Hawthorne effect).

Methods: We constructed a cohort of 3889 young women aged 11-20 years using data from the Agincourt Health and socio-Demographic Surveillance System. We compared school enrollment in 2011 (trial start) and 2015 (trial end) between those who did (n=1720) and did not (n=2169) enroll in the trial. To isolate the Hawthorne effect, we restricted the cohort to those enrolled in school in 2011.

Results: In 2011, trial participants were already more likely to be enrolled in school compared to non-participants [RD (95% CI): 6.3 (5.1, 7.5)]. Restricted to those enrolled in school in 2011, trial participants were also more likely to be enrolled in school in 2015 [RD (95% CI): 7.3 (4.5, 10.2)]. The strength of association increased with age.

Conclusions: Trial participants across both study arms were more likely to be enrolled in school than non-participants. Our findings suggest that both sample selection and Hawthorne effects may have diminished the differences in school enrollment between study arms, a plausible explanation for the null trial findings. The Hawthorne-specific findings generate hypotheses for how to structure school retention interventions to prevent HIV.

Key words: Hawthorne effect, selection effect, HIV prevention, adolescents, South Africa

For peer review only - http://bmjopen.bmj.com/site/about/guidelines.xhtml

Strengths and limitations of this study:

- To our knowledge, this study is the first to empirically examine whether Hawthorne effects may have influenced study results in an HIV prevention trial.
- We analyzed longitudinal data on a key study outcome (school enrollment) for the underlying population from which study participants were drawn. Complete data are not typically available for source populations in research studies.
- Our Hawthorne-specific findings suggest that aspects of the HPTN 068 protocol could potentially be adapted for school retention interventions to prevent HIV.
- It is important to note that data on HIV incidence, the primary endpoint of HPTN 068, were not available for the underlying target population.
- The differences we attribute to the Hawthorne effect were estimated in an observational dataset with adjustment for key sociodemographic characteristics. The potential for uncontrolled confounding requires that our results be interpreted cautiously.

For peer review only

INTRODUCTION

The evidence base for public health interventions largely comes from rigorous epidemiologic studies. However, results from epidemiologic studies may not be externally valid when study participant characteristics differ from those in the target population, even with randomization of exposure (referred to here as 'sample selection effect').[1] Further threats to validity can occur if study participation itself induces behavior change (Hawthorne effect, research participation effect, or trial effect, referred to here, collectively, as 'Hawthorne effect').[2,3] Analyzing study data to examine how results may have differed in the target population to which we would like to make inference is critical to making valid conclusions and policy recommendations.

Although epidemiologic training and research have long included at least cursory examinations of external validity,[4] with more recent methodological advancements around transportability of effect estimates from study populations to target populations,[5-7] empirical evaluation of Hawthorne effects is rare. The limited evidence for Hawthorne effects comes largely from clinical randomized controlled trials, most often assessed in cancer and nutrition studies,[8,9] with supporting evidence from HIV treatment research.[10] Trials designed to affect behavior change may be particularly susceptible to Hawthorne effects as the behaviors in question may be influenced by trial participation.[11,12] This is particularly true in HIV prevention research, where Hawthorne effects could pose validity threats if unexpectedly low HIV incidence occurs due to trial-induced risk behavior changes.[13] To our knowledge, no prior HIV prevention trial has empirically examined whether Hawthorne effects influenced study results.

In this study, we examine the potential influence of both sample selection effects and Hawthorne effects in the behavioral HIV Prevention Trial Network (HPTN) study 068,[14,15] designed to examine whether cash transfers conditional on school attendance reduce HIV acquisition in young South African women. Contrary to the study hypothesis, no difference in

For peer review only - http://bmjopen.bmj.com/site/about/guidelines.xhtml

HIV acquisition was observed between study arms, with high levels of school enrollment and low HIV incidence in both arms. These findings were surprising given the high background rates of school dropout in the study area[16-18] and the large body of evidence showing the positive impact of cash transfers on schooling outcomes[19], and limited the ability to explore schooling as a mechanism to reduce HIV risk.

Here, we contextualize HPTN 068 findings, using data on school enrollment in the underlying target population routinely collected by the Agincourt Health and socio-Demographic Surveillance System (HDSS) in which HPTN 068 was nested. We examine whether school enrollment trajectories of trial participants differed from non-participants, and whether differences could be attributed to existing differences in school enrollment at baseline (sample selection effect) or differences that arose during study participation (Hawthorne effect).

METHODS

Study setting and population

The Agincourt HDSS is located in the rural Bushbuckridge municipality of Mpumalanga province, South Africa[20] and has routinely collected annual vital event data on all people living in the study area since 1992. Other socio-demographic data is collected at regular but less frequent intervals. The Agincourt HDSS currently surveys the full cohort of over 115,000 people living across 31 villages, in an area of economic disadvantage with historically low access to public services. However, government schools in the study site are free and often provide feeding programs. HIV contributes a large burden to the community with 19% HIV prevalence overall in those aged 15 years and older.[21]

HPTN 068 was a Phase III individually-randomized trial designed to examine whether cash transfers conditional on school attendance influence the risk of HIV acquisition in young

For peer review only - http://bmjopen.bmj.com/site/about/guidelines.xhtml

women.[14 15] Key selection criteria for participation in the study were: current enrollment in grades 8-11; age 13-20 years; not married or pregnant at baseline; and having a caregiver with the documents necessary to open a bank account. Age-eligible young women were identified from Agincourt HDSS records to be contacted for further eligibility screening (n=10,134).[14] Between March 2011 and December 2012 a total of 2533 young women were enrolled. Participants were seen annually for a maximum of three years from enrollment or until high school graduation. Thus, participants who enrolled in the trial in 2011 in grade 11 could exit the study as early as 2012 after graduating high school. Participants who enrolled in the trial in 2012 in grade 8 or 9 could exit the study as late as March 2015 (Figure 1).

Community, household, and individual consents have been obtained for all Agincourt HDSS census research since its inception with informed verbal consent obtained at each census round. Ethical approval was obtained from the University of the Witwatersrand's Human Research Ethics Committee (updated # M110138; original # M960720) and the Mpumalanga Province Health Research Committee. Ethical approvals for HPTN 068 were provided by the Office of Human Research Ethics at the University of North Carolina-Chapel Hill (#10–1868), the University of the Witwatersrand's Human Research Ethics Committee (#101012), and the Mpumalanga Province Health Research Committee. Ethical approval for this analysis was provided by the Indiana University Office of Research Compliance (#1608116129).

For peer review only

Cohort construction

We constructed our analytic cohort to identify all young women living in the study area at the time of trial start (2011) regardless of trial participation status. Further restrictions were applied to build a cohort of young women on comparable age/grade trajectories and to match key HPTN 068 selection criteria. First, we restricted the cohort to include young women between the ages

of 13 and 20 years in 2011 or 2012. Based on education data collected by the Agincourt HDSS in 2009 (education data were collected in 2009 but not again until 2012), we also restricted the cohort to those who were enrolled in grades that projected to grades 8-11 in 2011 or 2012, assuming a one-grade increase each year.

Key measures

Our primary exposure of interest was *HPTN 068 trial participation* (both trial arms combined). Our primary outcome of interest was *school enrollment*, which we calculated at 2011 and 2015 based on Agincourt HDSS education data collected in 2009, 2012, and 2015. We used the 2011 school enrollment outcome to assess whether enrollment patterns were already different at the beginning of the trial, indicating a potential sample selection effect. We used the 2015 school enrollment outcome to assess whether enrollment patterns were different at the end of the trial, when both sample selection and Hawthorne effects could be present. We considered young women as enrolled in school if they indicated current school enrollment or if they reported a Grade 12 attainment, the final year of secondary schooling. The 2015 time-point aligned with a census education module, so enrollment status decisions were made based on data reported at that time. The 2011 time-point did not align with a census education module, so we inferred enrollment status at this time-point based on changes in status between 2009 and 2012. For example, if young women reported enrollment in both 2009 and 2012, we inferred they were also enrolled in 2011. If young women reported enrollment in 2009 but not 2012, we used changes in educational attainment to infer whether school dropout had occurred before or after 2011.

We also explored the potential for confounding and effect measure modification by key covariates. We examined age on January 1, 2011, categorized as ages of compulsory school enrollment (ages 11-15 years), older than age for compulsory school enrollment but correct age

For peer review only - http://bmjopen.bmj.com/site/about/guidelines.xhtml

for grade (ages 16-17 years), and older than age for compulsory school enrollment while also older than expected for grade (ages 18-20 years). We also examined indicators of household socio-economic status (SES), measured with a composite index of household assets; household size; gender of household head; secondary school educational attainment of the household head; country of origin (South African or Mozambican descent); and pre-2011 childbearing.

Analysis

We used binomial regression models with an identity link to estimate the association between trial participation and school enrollment at 2011 and 2015. The 2011 enrollment outcome was used to isolate the potential for a sample selection effect (i.e. Were trial participants more likely to be in school than non-participants at the beginning of the trial?). We used the 2015 enrollment outcome in a restricted cohort of young women who were enrolled in school in 2011 to isolate the potential for a Hawthorne effect at the end of the trial (i.e. Were trial participants more likely to remain in school after four years than non-participants, conditional on being in school at the beginning of the trial?).

We conducted unadjusted analyses and analyses adjusted for age, SES, gender and education of household head, household size, country of origin, and pre-2011 child-bearing. School enrollment decisions were likely highly influenced by age both because our cohort straddled the age limit for compulsory schooling in South Africa and because school dropout generally increases with age. Thus, we conducted age-stratified analyses to see whether the associations between trial participation and school enrollment differed by age category.

RESULTS

For peer review only - http://bmjopen.bmj.com/site/about/guidelines.xhtml

For peer review only

Overall 3889 young women from the Agincourt HDSS were included in our cohort (Table 1). Median age was 15 years (IQR: 14-16). Young women tended to live in large households (mean size: 8.4), and household heads were often female (42%) and often lacked high school education (86%). The majority of young women were of South African descent (60%) and very few (7%) had begun child-bearing prior to 2011. Just under half of the young women (44%) went on to participate in HPTN 068, and they tended to be less likely to be on the youngest (ages 11-12) or oldest (ages 19-20) end of the age spectrum, though median age in both participants and non-participants was 15. Trial participants were also less likely to have begun child-bearing, slightly more likely to live in households headed by high school graduates, and slightly more likely to live in household with above-median SES.

In 2011, at the time of HPTN 068 trial start, nearly everyone in the cohort was enrolled in school (96%), likely due to the age and 2009 enrollment requirements placed on the cohort (Table 2). However, young women who became trial participants were already more likely to be enrolled in school (99%) compared to non-participants (93%) [RD (95% CI): 6.3 (5.1, 7.5)], indicating a sample selection effect likely occurred as a consequence of the school enrollment eligibility criterion. Though the overall association attenuated after covariate adjustment [aRD (95% CI): 2.9 (-0.7, 6.5)], strong associations were observed in the 18-20 year old age group [aRD (95% CI): 19.5 (5.6, 33.3)], compared to the younger two age groups [aRD_{11-15} (95% CI): 0.4 (-5.2, 5.9); aRD_{16-17} (95% CI): 6.3 (0.5, 12.2)].

At the end of the trial in 2015, the difference in school enrollment between trial participants (81%) and non-participants (69%) grew, with an adjusted overall risk difference of 6.8 (95% CI: 3.4, 10.2). To investigate whether any differences in school enrollment could be attributed to a Hawthorne effect, we restricted the cohort to those enrolled in school in 2011 and examined differences in 2015. Under this restriction, young women enrolled in the trial were still more

For peer review only - http://bmjopen.bmj.com/site/about/guidelines.xhtml

likely to remain in school in 2015 (82%), compared to those who did not (74%) with an adjusted risk difference of 4.9 (95% CI: 1.5, 8.3)]. Again, the association was weakest among 11-15 year olds [aRD (95% CI): 1.8 (-1.3, 5.0)], and strongest among 18-20 year olds [aRD (95% CI): 22.8 (7.3, 38.2)].

DISCUSSION

HPTN 068 found that cash transfers conditional on school enrollment did not influence HIV acquisition among young women in a rural South African setting. Due to unexpectedly high levels of school enrollment in both arms, the ability to explore schooling as a mechanism through which cash transfers could influence HIV acquisition was limited. Here, we found evidence to suggest that both Hawthorne effects and sample selection effects could threaten the external validity of these findings. Overall, trial participants were more likely to remain in school until graduation than non-participants. Differences in school enrollment status were already apparent at the beginning of the study, suggesting that the trial selection criteria likely pulled in young women with better school enrollment behaviors than those who were not enrolled as trial participants (sample selection effect). Differences in school enrollment grew larger as the trial progressed, and, importantly, remained strong even after restricting to those enrolled in school in 2011 when the trial started, suggesting the changes in enrollment status occurred during the trial itself (Hawthorne effect). Both sample selection and Hawthorne effects may have diminished the differences in school enrollment between study arms and is one plausible explanation for the overall null study effect.

Our findings that trial participation influenced school enrollment behavior could plausibly be explained by several characteristics of the HPTN 068 study design and protocol. First, all participants were aware of the objective of the study: to retain young women in school to prevent HIV. This information could result in school enrollment behavior change to align with

For peer review only - http://bmjopen.bmj.com/site/about/guidelines.xhtml

perceived expectations of study staff or because young women were motivated to prevent HIV. Second, compared to non-participants, trial participants were exposed to different networks likely to be supportive of school enrollment. Adult fieldworkers showed interest in the schooling of participants with yearly in-person data collection and monthly in-school data collection. Data were collected in ‘camps’ wherein trial participants were transported to study offices annually for a half-day of surveys and blood tests, and entertaining activities during wait periods (e.g. fingernail painting, photograph taking, magazine reading). This protocol could have fostered a cohesive group environment among trial participants resulting in positive peer pressure to maintain school enrollment. There is a growing body of evidence that interventions providing a safe space with adult mentorship and peer support can have positive outcomes for young women in sub-Saharan Africa,[22-24] a pathway that may have been activated with trial participation. Finally, trial participation provided access to certain health and social services that may have otherwise been inaccessible, including annual HIV and HSV-2 testing and counseling, linkage to care for those who tested positive, and linkage to social work services for young women who reported experiences of sexual abuse. These services may have enabled young women who would have otherwise struggled with serious physical and mental health outcomes to remain in school.

Associations between trial participation and school enrollment were strongest in older age groups. The small differences observed in the youngest age group are understandable as they were under the age-limit of compulsory education with requirements to remain in school regardless of trial influence. For the oldest age group, trial selection criteria for lower grade levels meant they were older than expected for their grade, and suggested a history of grade repetition or temporary drop-out. That the trial protocol may have contributed to keeping older teens in school is significant as the transition to adulthood carries extremely high HIV risk.[21]

For peer review only

This study was fairly unusual in that data were available on key study outcomes for the underlying population from which study participants were drawn. The Agincourt HDSS routinely collects school enrollment data on all residents in the study area and we were able to leverage those data to assess differences between trial participants and non-participants. The majority of epidemiologic studies do not have the benefit of complete background data on the target population, and, as such, sample selection and Hawthorne effects are rarely empirically assessed.[3 8 10 12] However, when Hawthorne effects are assessed, the direction of the relationship between research participation and healthy outcomes tends to be positive, in line with our findings of improved school enrollment outcomes.

It is important to note that data on HIV incidence, the primary endpoint of HPTN 068, were not available for the underlying target population. We speculate that the improved schooling trajectories we observed in trial participants likely resulted in reduced risk of HIV acquisition.[15] Continued schooling is strongly associated with HIV prevention and reduced sexual risk outcomes in young women in sub-Saharan Africa[16 25 26] and we observed lower HIV incidence (1.8%) than expected among trial participants (3%). However, we cannot say with certainty that the association between trial participation and school enrollment extended to HIV protection.

The potential for uncontrolled confounding requires that our results be interpreted cautiously. The differences we attribute to the Hawthorne effect were estimated in an observational dataset. Initial screens for trial eligibility were performed based on age data maintained by the Agincourt HDSS and 82% of the eligible young women approached went on to enroll in the study, a fairly high response rate.[15] Still, it is plausible that those who refused participation were different from those who consented in ways that are also related to future school enrollment trajectories. Although we controlled for key socio-demographic characteristics that we theorized could be

For peer review only - http://bmjopen.bmj.com/site/about/guidelines.xhtml

related to both trial participation and school enrollment, the possibility for bias from unmeasured confounding remains.

We offer three key conclusions from this study. First, epidemiologists should give greater weight at the planning, analysis, and dissemination stages to identifying how sample selection and Hawthorne effects can be minimized, assessed, and discussed. Prioritizing research with well-defined target populations in areas with ongoing background data collection (e.g. HDSS centers) would improve researchers' abilities to empirically assess the external validity of their findings. Second, the sample selection effect we observed highlights how school-based samples can differ in important ways from non-school based samples in terms of underlying risk. Interventions focused on school-going adolescents may not reach those most in need of prevention, an anticipated issue that was ultimately difficult to avoid given HPTN 068 design. Third, the Hawthorne-specific findings suggest that aspects of the HPTN 068 protocol could potentially be adapted for school retention interventions to prevent HIV. If the relationship we observed is causal, the trial protocol increased school enrollment at a magnitude similar to targeted cash transfer interventions and other fairly resource-intensive school retention interventions in sub-Saharan Africa,[19 27-29] despite the actual contact with the young women being limited to annual visits. Future work should examine key elements of the study protocol – adult mentorship, peer support, school attendance monitoring, messaging around the link between school and HIV, routine HIV/STI testing and linkage to care – to better understand their relationship with school retention and HIV acquisition.

For peer review only - http://bmjopen.bmj.com/site/about/guidelines.xhtml

Acknowledgments: The Agincourt Health and socio-Demographic Surveillance System is supported by the Wellcome Trust (058893/Z/99/A; 069683/Z/02/Z; 085477/Z/08/Z; 085477/B/08/Z), the University of the Witwatersrand, and Medical Research Council, South Africa. HPTN 068 was supported by Award Numbers UM1 AI068619 (HPTN Leadership and Operations Center), UM1AI068617 (HPTN Statistical and Data Management Center), and UM1AI068613 (HPTN Laboratory Center) from the National Institute of Allergy and Infectious Diseases, the National Institute of Mental Health and the National Institute on Drug Abuse of the National Institutes of Health. This work was also supported by NIMH (R01MH087118) and the Carolina Population Center and its NIH Center grant (P2C HD050924). The content is solely the responsibility of the authors and does not necessarily represent the official views of the National Institutes of Health.

For peer review only

Figure 1. Timeline of Agincourt HDSS education data collection and HPTN 068 trial duration

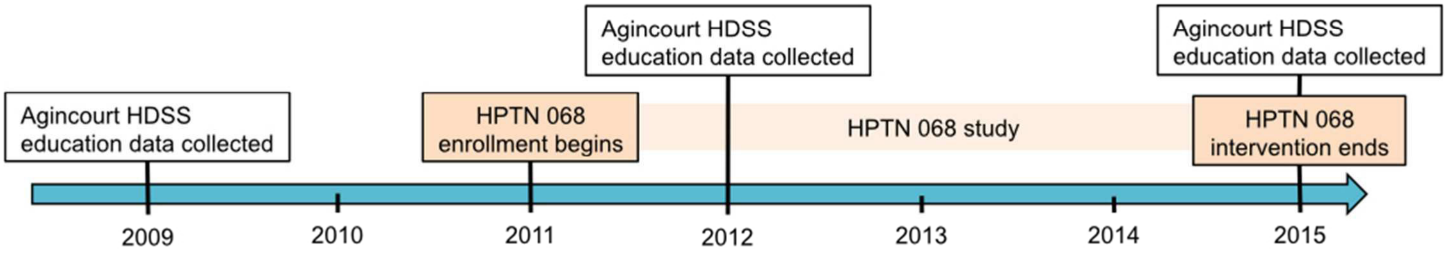

For peer review only

Table 1. Socio-demographic characteristics of a cohort of 3889 young women between the ages of 13-20 years in rural Agincourt, South Africa, by participation in the HPTN 068 trial

	Total (n=3889)		Trial participant[2] (n=1720)		Non-participant (n=2169)		
	N	%	N	%	N	%	p-value[1]
**Age in 2011**							<0.0001
11 - 12	188	4.8	26	1.5	162	7.5	
13 - 14	1270	32.7	637	37.0	633	29.2	
15 - 16	1591	40.9	793	46.1	798	36.8	
17 - 18	667	17.2	239	13.9	428	19.7	
19 - 20	173	4.5	25	1.5	148	6.8	
**SES***							0.003
At or above median	1778	50	853	52.7	925	47.8	
Below median	1778	50	766	47.3	1012	52.3	
Missing	333		101		232		
**Household size***							0.5
Mean	8.4		8.4		8.5		
SD	4.2		4.0		4.4		
Missing	46		14		32		
**Gender of HH head***							0.3
Female	1590	41.7	691	40.8	899	42.4	
Male	2224	58.3	1004	59.2	1220	57.6	
Missing	75		25		50		
**Household head educational attainment***							0.02
<Grade 12	3062	86.3	1398	87.8	1664	85.1	
Grad 12 or higher	486	13.7	194	12.2	292	14.9	
Missing	341		128		213		
**Country of origin***							0.2
South Africa	2315	59.6	1046	60.9	1269	58.6	
Mozambique	1570	40.4	673	39.2	897	41.4	
Missing	4		1		3		
**Childbearing before 2011**							<0.0001
Yes	274	7.1	67	3.9	207	9.5	
No	3615	93.0	1653	96.1	1962	90.5	
**2011 school enrollment**							<0.0001
Yes	3508	95.7	1637	99.2	1871	92.9	
No	158	4.3	14	0.9	144	7.2	
Missing	223		69		154		
**2015 school enrollment**							<0.0001
Yes	2465	74.2	1234	19	1231	68.5	
No	856	25.8	290	81	566	31.5	
Missing	568		196		372		

[1]p-values for categorical variables are from chi-square tests and for continuous variables are from t-tests

[2]Due to restrictions in the cohort construction to maintain comparable groups with respect to age and education status in 2009, not all of the 2533 HPTN 068 participants are represented.

*Measured in 2009

For peer review only - http://bmjopen.bmj.com/site/about/guidelines.xhtml

Table 2. The relationship between HPTN 068 trial participation and school enrollment in 2011 and 2015, stratified by age, in the full cohort and the cohort restricted to those enrolled in school in 2011

	Enrollment	N	Percent enrolled	RD (95% CI)	aRD[1] (95% CI)
2011: Full cohort					
**All ages**					
Trial participant	1637	1651	99.2 (98.7, 99.6)	6.3 (5.1, 7.5)	2.9 (-0.7, 6.5)
Non-trial participant	1871	2015	92.9 (91.7, 94.0)	1	1
**Ages 11-15**					
Trial participant	1036	1038	99.8 (99.5, 1.00)	0.6 (0.0, 1.2)	0.4 (-5.2, 5.9)
Non-trial participant	1115	1124	99.2 (98.6, 99.7	1	1
**Ages 16-17**					
Trial participant	532	539	98.7 (97.8, 99.7)	6.9 (4.6, 9.2)	6.3 (0.5, 12.2)
Non-trial participant	593	646	91.8 (89.7, 93.9)	1	1
**Ages 18-20**					
Trial participant	69	74	93.2 (87.5, 99.0)	26.7 (18.5, 34.9)	19.5 (5.6, 33.3)
Non-trial participant	163	245	66.5 (60.6, 72.4)	1	1
2015: Full cohort					
**All ages**					
Trial participant	1234	1524	81.0 (79.0, 83.0)	12.5 (9.6, 15.4)	6.8 (3.4, 10.2)
Non-trial participant	1231	1797	68.5 (66.4, 70.7)	1	1
**Ages 11-15**					
Trial participant	837	952	87.9 (85.9, 90.0)	3.8 (0.1, 6.9)	1.9 (-1.3, 5.0)
Non-trial participant	833	990	84.1 (81.9, 86.5)	1	1
**Ages 16-17**					
Trial participant	361	503	71.8 (67.9, 75.8)	12.5 (6.9, 18.1)	12.7 (6.7, 18.7)
Non-trial participant	346	584	59.3 (55.4, 63.4)	1	1
**Ages 18-20**					
Trial participant	36	69	52.2 (41.6, 65.4)	28.9 (15.8, 41.9)	29.7 (16.1, 43.3)
Non-trial participant	52	223	23.3 (18.4, 29.6)	1	1
2015: Restricted cohort[2]					
**All ages**					
Trial participant	1234	1510	81.7 (79.8, 83.7)	7.3 (4.5, 10.2)	4.9 (1.5, 8.3)
Non-trial participant	1231	1655	74.4 (72.3, 76.5)	1	1
**Ages 11-15**					
Trial participant	837	950	88.1 (86.1, 90.2)	3.2 (0.2, 6.3)	1.8 (-1.3, 5.0)
Non-trial participant	833	982	84.8 (82.6, 87.1)	1	1
**Ages 16-17**					
Trial participant	361	496	72.8 (69.0, 76.8)	7.6 (1.9, 13.3)	7.9 (1.9, 14.0)
Non-trial participant	346	531	65.2 (61.2, 69.3)	1	1
**Ages 18-20**					
Trial participant	36	64	56.3 (45.3, 69.8)	19.6 (5.1, 34.1)	22.8 (7.3, 38.2)
Non-trial participant	52	142	36.6 (29.5, 45.5)	1	1

[1]Adjusted for socio-economic status (coded dichotomously at median household asset index score), country of origin (South African versus Mozambican), educational attainment of household head (coded dichotomously at Grade 12 attainment), gender of household head (male versus female), household size (coded linearly), and pre-2011 childbearing (yes versus no). Models that are not age-stratified are also adjusted for age coded in two-year categories.

[2]Restricted to all young women who were enrolled in school in 2011

RD=risk difference; aRD=adjusted risk difference; CI=confidence interval

For peer review only

References

1. Westreich D, Edwards JK, Rogawski ET, et al. Causal impact: epidemiological approaches for a public health of consequence. *American journal of public health* 2016;106(6):1011.
2. Adair JG. The Hawthorne effect: A reconsideration of the methodological artifact. *Journal of applied psychology* 1984;69(2):334.
3. McCarney R, Warner J, Iliffe S, et al. The Hawthorne Effect: a randomised, controlled trial. *BMC medical research methodology* 2007;7(1):1.
4. Steckler A, McLeroy KR. The importance of external validity: American Public Health Association, 2008.
5. Cole SR, Stuart EA. Generalizing evidence from randomized clinical trials to target populations the actg 320 trial. *American journal of epidemiology* 2010;172(1):107-15.
6. Hernán MA, VanderWeele TJ. Compound treatments and transportability of causal inference. *Epidemiology (Cambridge, Mass)* 2011;22(3):368.
7. Westreich D, Edwards JK. Invited commentary: every good randomization deserves observation. *American journal of epidemiology* 2015;182(10):857-60.
8. Braunholtz DA, Edwards SJ, Lilford RJ. Are randomized clinical trials good for us (in the short term)? Evidence for a “trial effect”. *Journal of clinical epidemiology* 2001;54(3):217-24.
9. Robinson E, Hardman CA, Halford JC, et al. Eating under observation: a systematic review and meta-analysis of the effect that heightened awareness of observation has on laboratory measured energy intake. *The American journal of clinical nutrition* 2015 doi: 10.3945/ajcn.115.111195 [published Online First: 2015/07/17]
10. Menezes P, Miller WC, Wohl DA, et al. Does HAART efficacy translate to effectiveness? Evidence for a trial effect. *PloS one* 2011;6(7):e21824.
11. McCambridge J, Kypri K, Elbourne D. In randomization we trust? There are overlooked problems in experimenting with people in behavioral intervention trials. *J Clin Epidemiol* 2014;67(3):247-53. doi: 10.1016/j.jclinepi.2013.09.004 [published Online First: 2013/12/10]
12. McCambridge J, Witton J, Elbourne DR. Systematic review of the Hawthorne effect: new concepts are needed to study research participation effects. *J Clin Epidemiol* 2014;67(3):267-77. doi: 10.1016/j.jclinepi.2013.08.015 [published Online First: 2013/11/28]
13. Padian NS, McLOY SI, Balkus JE, et al. Weighing the gold in the gold standard: challenges in HIV prevention research. *AIDS (London, England)* 2010;24(5):621.
14. Pettifor A, MacPhail C, Selin A, et al. HPTN 068: a randomized control trial of a conditional cash transfer to reduce HIV infection in young women in South Africa—study design and baseline results. *AIDS and behavior* 2016;20(9):1863-82.
15. Pettifor A, MacPhail C, Hughes JP, et al. The effect of a conditional cash transfer on HIV incidence in young women in rural South Africa (HPTN 068): a phase 3, randomised controlled trial. *The Lancet Global Health* 2016;4(12):e978-e88.
16. Rosenberg M, Pettifor A, Miller WC, et al. Relationship between school dropout and teen pregnancy among rural South African young women. *International journal of epidemiology* 2015:dyv007.
17. Strassburg S, Meny-Gibert S, Russell B. Left unfinished: Temporary absence and drop-out from South African schools. Access to Education. Johannesburg, South Africa: Social Surveys Africa, 2010.

For peer review only

18. Department of Basic Education Republic of South Africa. Education for All 2014 Progress Report: South Africa. Pretoria, 2014.
19. Baird S, Ferreira FH, Özler B, et al. Relative effectiveness of conditional and unconditional cash transfers for schooling outcomes in developing countries: a systematic review. *Campbell systematic reviews* 2013;9(8)
20. Kahn K, Collinson MA, Gomez-Olive FX, et al. Profile: Agincourt Health and Socio-demographic Surveillance System. *International journal of epidemiology* 2012;41(4):988-1001. doi: 10.1093/ije/dys115 [published Online First: 2012/08/31]
21. Gómez-Olivé FX, Angotti N, Houle B, et al. Prevalence of HIV among those 15 and older in rural South Africa. *AIDS care* 2013;25(9):1122-28.
22. Erulkar AS, Muthengi E. Evaluation of Berhane Hewan: a program to delay child marriage in rural Ethiopia. *International Perspectives on Sexual and Reproductive Health* 2009:6-14.
23. Bandiera O, Buehren N, Burgess R, et al. Empowering adolescent girls: Evidence from a randomized control trial in Uganda. *Washington, DC: World Bank* 2012
24. Austrian K, Hewett P, Soler-Hampejsek E, et al. Adolescent Girls Empowerment Programme: Research and Evaluation Mid-Term Technical Report. Lusaka, Zambia: Population Council, 2016.
25. Pettifor AE, Levandowski BA, MacPhail C, et al. Keep them in school: the importance of education as a protective factor against HIV infection among young South African women. *International journal of epidemiology* 2008;37(6):1266-73. doi: 10.1093/ije/dyn131 [published Online First: 2008/07/11]
26. Jukes M, Simmons S, Bundy D. Education and vulnerability: the role of schools in protecting young women and girls from HIV in southern Africa. *AIDS (London, England)* 2008;22 Suppl 4:S41-56. doi: 10.1097/01.aids.0000341776.71253.04 [published Online First: 2008/12/17]
27. Cho H, Hallfors DD, Mbai, II, et al. Keeping adolescent orphans in school to prevent human immunodeficiency virus infection: evidence from a randomized controlled trial in Kenya. *The Journal of adolescent health : official publication of the Society for Adolescent Medicine* 2011;48(5):523-6. doi: 10.1016/j.jadohealth.2010.08.007 [published Online First: 2011/04/20]
28. Hallfors D, Cho H, Rusakaniko S, et al. Supporting adolescent orphan girls to stay in school as HIV risk prevention: evidence from a randomized controlled trial in Zimbabwe. *Am J Public Health* 2011;101(6):1082-8. doi: AJPH.2010.300042 [pii]10.2105/AJPH.2010.300042 [published Online First: 2011/04/16]
29. Baird S, Chirwa E, McIntosh C, et al. The short-term impacts of a schooling conditional cash transfer program on the sexual behavior of young women. *Health economics* 2010;19 Suppl:55-68. doi: 10.1002/hec.1569 [published Online First: 2009/12/01]

For peer review only - http://bmjopen.bmj.com/site/about/guidelines.xhtml

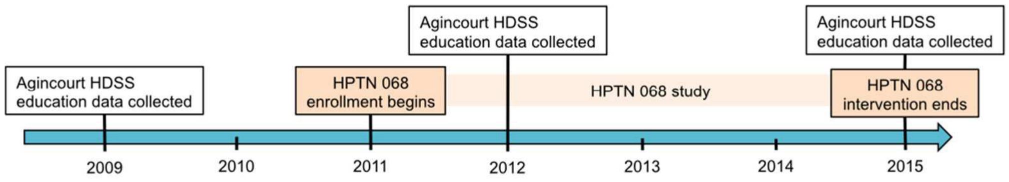

361x270mm (72 x 72 DPI)

review only

STROBE Statement—Checklist of items that should be included in reports of ***cohort studies***

	Item No	Recommendation	Page number
**Title and abstract**	1	(*a*) Indicate the study's design with a commonly used term in the title or the abstract	2
		(*b*) Provide in the abstract an informative and balanced summary of what was done and what was found	2
**Introduction**			
Background/rationale	2	Explain the scientific background and rationale for the investigation being reported	4
Objectives	3	State specific objectives, including any prespecified hypotheses	5
**Methods**			
Study design	4	Present key elements of study design early in the paper	5-7
Setting	5	Describe the setting, locations, and relevant dates, including periods of recruitment, exposure, follow-up, and data collection	5-7
Participants	6	(*a*) Give the eligibility criteria, and the sources and methods of selection of participants. Describe methods of follow-up	5-7
		(*b*) For matched studies, give matching criteria and number of exposed and unexposed	
Variables	7	Clearly define all outcomes, exposures, predictors, potential confounders, and effect modifiers. Give diagnostic criteria, if applicable	7
Data sources/ measurement	8*	For each variable of interest, give sources of data and details of methods of assessment (measurement). Describe comparability of assessment methods if there is more than one group	*7*
Bias	9	Describe any efforts to address potential sources of bias	8
Study size	10	Explain how the study size was arrived at	6-7
Quantitative variables	11	Explain how quantitative variables were handled in the analyses. If applicable, describe which groupings were chosen and why	7-8
Statistical methods	12	(*a*) Describe all statistical methods, including those used to control for confounding	8
		(*b*) Describe any methods used to examine subgroups and interactions	8
		(*c*) Explain how missing data were addressed	Table 1
		(*d*) If applicable, explain how loss to follow-up was addressed	Table 1
		(*e*) Describe any sensitivity analyses	n/a
**Results**			
Participants	13*	(a) Report numbers of individuals at each stage of study—eg numbers potentially eligible, examined for eligibility, confirmed eligible, included in the study, completing follow-up, and analysed	9
		(b) Give reasons for non-participation at each stage	n/a
		(c) Consider use of a flow diagram	n/a
Descriptive data	14*	(a) Give characteristics of study participants (eg demographic, clinical, social) and information on exposures and potential confounders	9
		(b) Indicate number of participants with missing data for each variable of interest	Table 1
		(c) Summarise follow-up time (eg, average and total amount)	n/a
Outcome data	15*	Report numbers of outcome events or summary measures over time	Table 1
Main results	16	(*a*) Give unadjusted estimates and, if applicable, confounder-adjusted estimates and their precision (eg, 95% confidence interval). Make clear	Table 2

For peer review only

		which confounders were adjusted for and why they were included	
		(*b*) Report category boundaries when continuous variables were categorized	Table 2
		(*c*) If relevant, consider translating estimates of relative risk into absolute risk for a meaningful time period	Table 2
Other analyses	17	Report other analyses done—eg analyses of subgroups and interactions, and sensitivity analyses	Table 2
**Discussion**			
Key results	18	Summarise key results with reference to study objectives	10
Limitations	19	Discuss limitations of the study, taking into account sources of potential bias or imprecision. Discuss both direction and magnitude of any potential bias	10-13
Interpretation	20	Give a cautious overall interpretation of results considering objectives, limitations, multiplicity of analyses, results from similar studies, and other relevant evidence	10-13
Generalisability	21	Discuss the generalisability (external validity) of the study results	10-13
**Other information**			
Funding	22	Give the source of funding and the role of the funders for the present study and, if applicable, for the original study on which the present article is based	14

*Give information separately for exposed and unexposed groups.

Note: An Explanation and Elaboration article discusses each checklist item and gives methodological background and published examples of transparent reporting. The STROBE checklist is best used in conjunction with this article (freely available on the Web sites of PLoS Medicine at http://www.plosmedicine.org/, Annals of Internal Medicine at http://www.annals.org/, and Epidemiology at http://www.epidem.com/). Information on the STROBE Initiative is available at http://www.strobe-statement.org.

For peer review only

For peer review only - http://bmjopen.bmj.com/site/about/guidelines.xhtml

BMJ Open

Evidence for sample selection effect and Hawthorne effect in behavioral HIV prevention trial among young women in a rural South African community

Journal:	*BMJ Open*
Manuscript ID	bmjopen-2017-019167.R1
Article Type:	Research
Date Submitted by the Author:	02-Nov-2017

Complete List of Authors:	Rosenberg, Molly; Indiana University School of Public Health-Bloomington, Epidemiology and Biostatistics<br>Pettifor, Audrey; University of North Carolina at Chapel Hill, Department of Epidemiology, School of Public Health<br>Twine, Rhian; University of the Witwatersrand, MRC/Wits Rural Public Health and Health Transitions Research Unit (Agincourt), School of Public Health, Faculty of Health Sciences<br>Hughes, James; University of Washington School of Public Health, Biostatistics<br>Gómez-Olivé, F. Xavier; University of the Witwatersrand, Medical Research Council/Wits Rural Health and Health Transitions Unit (Agincourt), School of Public Health, Faculty of Health Sciences<br>Wagner, Ryan; University of the Witwatersrand, Johannesburg, MRC/Wits Rural Public Health & Health Transitions Research Unit (Agincourt)<br>Sulaimon, Afolabi; University of the Witwatersrand, 5. MRC/Wits Rural Public Health and Health Transitions Research Unit (Agincourt)<br>Tollman, Stephen; University of the Witwatersrand, School of Public Health, Faculty of Health Sciences<br>Selin, Amanda; University of North Carolina Chapel Hill, Carolina Population Center<br>MacPhail, Catherine; University of the Witwatersrand School of Clinical Medicine, Wits Reproductive Health and HIV Institute (WRHI); University of New England, School of Health<br>Kahn, Kathleen; University of the Witwatersrand, Medical Research Council/Wits Rural Health and Health Transitions Unit (Agincourt), School of Public Health, Faculty of Health Sciences

**Primary Subject Heading**:	Epidemiology
Secondary Subject Heading:	Global health, HIV/AIDS, Research methods
Keywords:	Hawthorne effect, selection effect, HIV prevention, adolescents, South Africa

SCHOLARONE™
Manuscripts

For peer review only

Title: Evidence for sample selection effect and Hawthorne effect in behavioral HIV prevention trial among young women in a rural South African community

Authors: Molly Rosenberg[1,2], Audrey Pettifor[3,4,5], Rhian Twine[5], James P. Hughes[6], F. Xavier Gomez-Olive[2,5,7], Ryan G. Wagner[5,10], Afolabi Sulaimon[5], Stephen Tollman[5,7,10], Amanda Selin[4], Catherine Macphail[5,8,9], Kathleen Kahn[5,7,10]

Author affiliations:
1. Department of Epidemiology and Biostatistics, Indiana University School of Public Health-Bloomington
2. Center for Population and Development Studies, Harvard University
3. Department of Epidemiology, University of North Carolina-Chapel Hill
4. Carolina Population Center, University of North Carolina-Chapel Hill
5. MRC/Wits Rural Public Health and Health Transitions Research Unit (Agincourt), School of Public Health, Faculty of Health Sciences, University of the Witwatersrand, Johannesburg, South Africa
6. Department of Biostatistics, University of Washington
7. INDEPTH Network, Accra, Ghana
8. School of Health and Society, University of Wollongong, New South Wales, Australia
9. Wits Reproductive Health and HIV Institute, University of the Witwatersrand, Johannesburg, South Africa
10. Umeå Centre for Global Health Research, Division of Epidemiology and Global Health, Department of Public Health and Clinical Medicine, Umeå University, Umeå, Sweden

Corresponding author:
Molly Rosenberg
1025 E. 7th Street, Office C032
Bloomington, IN 47405
Email: rosenmol@indiana.edu
Phone: +1 (812) 856-2509
Fax: +1 (812) 855-9107

Word count: 3445/3000

Author contributions: MR, AP, RT, and KK conceived the study. MR conducted the analysis and wrote the first draft of the manuscript. AP, RT, JPH, FXGO, RGW, AS, ST, AS, CM, and KK were involved in the design of the parent studies and/or in collection, storage, and analysis of data from the parent study. All authors contributed to the interpretation of the findings, critical review of the manuscript, and approval of the final manuscript as submitted.

Data sharing statement: Agincourt HDSS data access can be requested at the following link: http://www.agincourt.co.za/index.php/data/

Competing interests: The authors declare no competing interests.

For peer review only

Abstract (262/300 words)

Objectives: We examined the potential influence of both sample selection effects and Hawthorne effects in the behavioral HIV Prevention Trial Network (HPTN) study 068, designed to examine whether cash transfers conditional on school attendance reduce HIV acquisition in young South African women. We explored whether school enrollment among study participants differed from the underlying population, and whether differences existed at baseline (sample selection effect) or arose during study participation (Hawthorne effect).

Methods: We constructed a cohort of 3889 young women aged 11-20 years using data from the Agincourt Health and socio-Demographic Surveillance System. We compared school enrollment in 2011 (trial start) and 2015 (trial end) between those who did (n=1720) and did not (n=2169) enroll in the trial. To isolate the Hawthorne effect, we restricted the cohort to those enrolled in school in 2011.

Results: In 2011, trial participants were already more likely to be enrolled in school (99%) compared to non-participants (93%). However, this association was attenuated with covariate adjustment [aRD (95% CI): 2.9 (-0.7, 6.5)]. Restricting to those enrolled in school in 2011, trial participants were also more likely to be enrolled in school in 2015 [aRD (95% CI): 4.9 (1.5, 8.3)]. The strength of associations increased with age.

Conclusions: Trial participants across both study arms were more likely to be enrolled in school than non-participants. Our findings suggest that both sample selection and Hawthorne effects may have diminished the differences in school enrollment between study arms, a plausible explanation for the null trial findings. The Hawthorne-specific findings generate hypotheses for how to structure school retention interventions to prevent HIV.

For peer review only

For peer review only - http://bmjopen.bmj.com/site/about/guidelines.xhtml

Key words: Hawthorne effect, selection effect, HIV prevention, adolescents, South Africa

Strengths and limitations of this study:

- To our knowledge, this study is the first to empirically examine whether Hawthorne effects may have influenced study results in an HIV prevention trial.
- We analyzed longitudinal data on a key study outcome (school enrollment) for the underlying population from which study participants were drawn. Complete data are not typically available for source populations in research studies.
- Our Hawthorne-specific findings suggest that aspects of the HPTN 068 protocol could potentially be adapted for school retention interventions to prevent HIV.
- It is important to note that data on HIV incidence, the primary endpoint of HPTN 068, were not available for the underlying target population.
- The differences we attribute to the Hawthorne effect were estimated in an observational dataset with adjustment for key sociodemographic characteristics. The potential for uncontrolled confounding requires that our results be interpreted cautiously.

For peer review only

INTRODUCTION

The evidence base for public health interventions largely comes from rigorous epidemiologic studies.[1 2] However, results from epidemiologic studies may not be generalizable (or 'externally valid') when study participant characteristics differ from those in the target population, even with randomization of exposure (referred to here as 'sample selection effect').[3] Further threats to validity can occur if study participation itself induces behavior change (Hawthorne effect, research participation effect, or trial effect, referred to here, collectively, as 'Hawthorne effect').[4 5] Analyzing study data to examine how results may have differed in the target population to which we would like to make inference is critical to making valid conclusions and policy recommendations.

Although epidemiologic training and research have long included at least cursory examinations of external validity,[6] with more recent methodological advancements around transportability of effect estimates from study populations to target populations,[7-9] empirical evaluation of Hawthorne effects is rare. The limited evidence for Hawthorne effects comes largely from clinical randomized controlled trials, most often assessed in cancer and nutrition studies,[10 11] with supporting evidence from HIV treatment research.[12] Trials designed to affect behavior change may be particularly susceptible to Hawthorne effects as the behaviors in question may be influenced by trial participation.[13 14] This is particularly true in HIV prevention research, where Hawthorne effects could pose validity threats if unexpectedly low HIV incidence occurs due to trial-induced risk behavior changes.[15] To our knowledge, no prior HIV prevention trial has empirically examined whether Hawthorne effects influenced study results.

In this study, we examine the potential influence of both sample selection effects and Hawthorne effects in the behavioral HIV Prevention Trial Network (HPTN) study 068,[16 17] designed to examine whether cash transfers conditional on school attendance reduce HIV

For peer review only - http://bmjopen.bmj.com/site/about/guidelines.xhtml

acquisition in young South African women. Contrary to the study hypothesis, no difference in HIV acquisition was observed between study arms, with high levels of school enrollment and low HIV incidence in both arms. These findings were surprising given the high background rates of school dropout in the study area[18-20] and the large body of evidence showing the positive impact of cash transfers on schooling outcomes[21], and limited the ability to explore schooling as a mechanism to reduce HIV risk.

Here, we contextualize HPTN 068 findings, using data on school enrollment in the underlying target population routinely collected by the Agincourt Health and socio-Demographic Surveillance System (HDSS) in which HPTN 068 was nested. We examine whether school enrollment trajectories of trial participants differed from non-participants, and whether differences could be attributed to existing differences in school enrollment at baseline (sample selection effect) or differences that arose during study participation (Hawthorne effect).

METHODS

Study setting and population

The Agincourt HDSS is located in the rural Bushbuckridge municipality of Mpumalanga province, South Africa[22] and has routinely collected annual vital event data on all people living in the study area since 1992. Other socio-demographic data are collected at regular but less frequent intervals. For example, educational attainment is queried every three years, employment data are collected every four years, and a household asset index is measured every other year. The Agincourt HDSS currently surveys the full cohort of over 115,000 people living across 31 villages, in an area of economic disadvantage with historically low access to public services. However, government schools in the study site are free and often provide feeding programs. HIV contributes a large burden to the community with 19% HIV prevalence overall in those aged 15 years and older.[23]

For peer review only - http://bmjopen.bmj.com/site/about/guidelines.xhtml

HPTN 068 was a Phase III individually-randomized trial designed to examine whether cash transfers conditional on school attendance influence the risk of HIV acquisition in young women.[16 17] Young women and their caregivers were randomly assigned to receive a monthly cash transfer conditional on ≥80% school attendance or no cash transfer. The size of the monthly cash transfer was 300 Rands (R; about US$30 in 2012), and was divided into R200 provided to the caregiver and R100 provided to the young woman. Key selection criteria for participation in the study were: current enrollment in grades 8-11; age 13-20 years; not married or pregnant at baseline; and having a caregiver with the documents necessary to open a bank account. Age-eligible young women were identified from Agincourt HDSS records to be contacted for further eligibility screening (n=10,134).[16] Between March 2011 and December 2012 a total of 2533 young women were enrolled. Participants were seen annually for a maximum of three years from enrollment or until high school graduation. Thus, participants who enrolled in the trial in 2011 in grade 11 could exit the study as early as 2012 after graduating high school. Participants who enrolled in the trial in 2012 in grade 8 or 9 could exit the study as late as March 2015 (Figure 1).

Community, household, and individual consents have been obtained for all Agincourt HDSS census research since its inception with informed verbal consent obtained at each census round. Ethical approval was obtained from the University of the Witwatersrand's Human Research Ethics Committee (updated # M110138; original # M960720) and the Mpumalanga Province Health Research Committee. Ethical approvals for HPTN 068 were provided by the Office of Human Research Ethics at the University of North Carolina-Chapel Hill (#10–1868), the University of the Witwatersrand's Human Research Ethics Committee (#101012), and the

For peer review only - http://bmjopen.bmj.com/site/about/guidelines.xhtml

Mpumalanga Province Health Research Committee. Ethical approval for this analysis was provided by the Indiana University Office of Research Compliance (#1608116129).

Cohort construction

We constructed our analytic cohort to identify all young women living in the study area at the time of trial start (2011) regardless of trial participation status. Further restrictions were applied to build a cohort of young women on comparable age/grade trajectories and to match key HPTN 068 selection criteria. First, we restricted the cohort to include young women between the ages of 13 and 20 years in 2011 or 2012. Based on education data collected by the Agincourt HDSS in 2009 (education data were collected in 2009 but not again until 2012), we also restricted the cohort to those who were enrolled in grades that projected to grades 8-11 in 2011 or 2012, assuming a one-grade increase each year.

Key measures

Our primary exposure of interest was *HPTN 068 trial participation* (both trial arms combined). We analyzed both arms together because trial results indicated essentially no differences in school attendance and enrollment data between the arms. School attendance was high (≥80%) for 95% of the intervention arm and 96% of the control arm participants. Permanent school dropout occurred at a rate of 3 per 100 person years in both arms.[17]

Our primary outcome of interest was *school enrollment*, which we calculated at 2011 and 2015 based on Agincourt HDSS education data collected in 2009, 2012, and 2015. We used the 2011 school enrollment outcome to assess whether enrollment patterns were already different at the beginning of the trial, indicating a potential sample selection effect. We used the 2015 school enrollment outcome to assess whether enrollment patterns were different at the end of the trial, when both sample selection and Hawthorne effects could be present. We considered young

For peer review only - http://bmjopen.bmj.com/site/about/guidelines.xhtml

women as enrolled in school if they indicated current school enrollment or if they reported a Grade 12 attainment, the final year of secondary schooling.

With the 2009, 2012, and 2015 education modules, fieldworkers updated the highest education level each young woman achieved and recorded whether or not she was currently enrolled in school. The 2015 time-point aligned with a census education module, so enrollment status decisions were made based on data reported at that time. The 2011 time-point did not align with a census education module, so we inferred enrollment status at this time-point based on changes in status between 2009 and 2012. If young women reported enrollment in both 2009 and 2012, we inferred they were also enrolled in 2011. If young women reported enrollment in 2009 but not 2012, we used changes in educational attainment to infer whether school dropout had occurred before or after 2011. For example, if a young woman reported grade 7 attainment in 2009 and grade 10 attainment in 2012, we assumed that the three years of additional education were accumulated in 2009, 2010, and 2011. This young woman would be coded as enrolled in school in 2011. If a young woman reported grade 7 attainment in 2009 and grade 9 attainment in 2012, we assumed that the two years of additional education were accumulated in 2009 and 2010. This young woman would be coded as not enrolled in school in 2011. Observations with illogical education data patterns between 2009 and 2012 (e.g. educational attainment decreases over time) were coded as missing for school enrollment in 2011.

For peer review only

We also explored the potential for confounding and effect measure modification by key covariates. We examined age on January 1, 2011, categorized as ages of compulsory school enrollment (ages 11-15 years), older than age for compulsory school enrollment but correct age for grade (ages 16-17 years), and older than age for compulsory school enrollment while also older than expected for grade (ages 18-20 years). We also examined indicators of household socio-economic status (SES), measured with a composite index of household assets;

For peer review only - http://bmjopen.bmj.com/site/about/guidelines.xhtml

household size; gender of household head; secondary school educational attainment of the household head; country of origin (South African or Mozambican descent); and pre-2011 childbearing.

Analysis

We used binomial regression models with an identity link to estimate the association between trial participation and school enrollment at 2011 and 2015. The 2011 enrollment outcome was used to isolate the potential for a sample selection effect (i.e. Were trial participants more likely to be in school than non-participants at the beginning of the trial?). We used the 2015 enrollment outcome in a restricted cohort of young women who were enrolled in school in 2011 to isolate the potential for a Hawthorne effect at the end of the trial (i.e. Were trial participants more likely to remain in school after four years than non-participants, conditional on being in school at the beginning of the trial?).

We conducted unadjusted analyses and analyses adjusted for age, SES, gender and education of household head, household size, country of origin, and pre-2011 child-bearing. School enrollment decisions were likely highly influenced by age both because our cohort straddled the age limit for compulsory schooling in South Africa and because school dropout generally increases with age. Thus, we conducted age-stratified analyses to see whether the associations between trial participation and school enrollment differed by age category.

Although trial results indicated that school attendance and enrollment outcomes were not significantly different between arms of the trial, the intervention was designed to incentivize school attendance.[17] For this reason, we conducted a sensitivity analysis restricting the trial participants to those who were randomly assigned to the control group only. We compared results from this restricted population to results from analysis of the full trial population.

For peer review only - http://bmjopen.bmj.com/site/about/guidelines.xhtml

RESULTS

Overall 3889 young women from the Agincourt HDSS were included in our cohort (Table 1). Median age was 15 years (IQR: 14-16). Young women tended to live in large households (mean size: 8.4), and household heads were often female (42%) and often lacked high school education (86%). The majority of young women were of South African descent (60%) and very few (7%) had begun child-bearing prior to 2011. Just under half of the young women (44%) went on to participate in HPTN 068, and they tended to be less likely to be on the youngest (ages 11-12) or oldest (ages 19-20) end of the age spectrum, though median age in both participants and non-participants was 15. Trial participants were also less likely to have begun child-bearing, slightly more likely to live in households headed by high school graduates, and slightly more likely to live in household with above-median SES.

In 2011, at the time of HPTN 068 trial start, nearly everyone in the cohort was enrolled in school (96%), likely due to the age and 2009 enrollment requirements placed on the cohort (Table 2). However, young women who became trial participants were already more likely to be enrolled in school (99%) compared to non-participants (93%) [RD (95% CI): 6.3 (5.1, 7.5)], indicating a sample selection effect likely occurred as a consequence of the school enrollment eligibility criterion. Though the overall association attenuated after covariate adjustment [aRD (95% CI): 2.9 (-0.7, 6.5)], strong associations were observed in the 18-20 year old age group [aRD (95% CI): 19.5 (5.6, 33.3)], compared to the younger two age groups [aRD_{11-15} (95% CI): 0.4 (-5.2, 5.9); aRD_{16-17} (95% CI): 6.3 (0.5, 12.2)].

At the end of the trial in 2015, the difference in school enrollment between trial participants (81%) and non-participants (69%) grew, with an adjusted overall risk difference of 6.8 (95% CI: 3.4, 10.2). To investigate whether any differences in school enrollment could be attributed to a

For peer review only - http://bmjopen.bmj.com/site/about/guidelines.xhtml

Hawthorne effect, we restricted the cohort to those enrolled in school in 2011 and examined differences in 2015. Under this restriction, young women enrolled in the trial were still more likely to remain in school in 2015 (82%), compared to those who did not (74%) with an adjusted risk difference of 4.9 (95% CI: 1.5, 8.3)]. Again, the association was weakest among 11-15 year olds [aRD (95% CI): 1.8 (-1.3, 5.0)], and strongest among 18-20 year olds [aRD (95% CI): 22.8 (7.3, 38.2)].

Results were largely unchanged when we restricted the trial participant population to those enrolled in the control group only (Table 3). Although confidence intervals widened due to reduced sample size with some newly spanning the null, the magnitudes of the risk difference point estimates were largely unchanged from those in the primary analysis.

DISCUSSION

HPTN 068 found that cash transfers conditional on school enrollment did not influence HIV acquisition among young women in a rural South African setting. Due to unexpectedly high levels of school enrollment in both arms, the ability to explore schooling as a mechanism through which cash transfers could influence HIV acquisition was limited. Here, we found evidence to suggest that both Hawthorne effects and sample selection effects could threaten the external validity of these findings. Overall, trial participants were more likely to remain in school until graduation than non-participants. Differences in school enrollment status were already apparent at the beginning of the study, suggesting that the trial selection criteria likely pulled in young women with better school enrollment behaviors than those who were not enrolled as trial participants (sample selection effect). Differences in school enrollment grew larger as the trial progressed, and, importantly, remained strong even after restricting to those enrolled in school in 2011 when the trial started, suggesting the changes in enrollment status occurred during the trial itself (Hawthorne effect). Both sample selection and Hawthorne effects

For peer review only

may have diminished the differences in school enrollment between study arms and is one plausible explanation for the overall null study effect. The HPTN 068 trial was designed to activate the HIV prevention effects of education by incentivizing school attendance and retention in the intervention arm. With high levels of school attendance and retention across both arms of the trial, the ability to detect a trial effect was likely weakened.

Our findings that trial participation influenced school enrollment behavior could plausibly be explained by several characteristics of the HPTN 068 study design and protocol. First, all participants were aware of the objective of the study: to retain young women in school to prevent HIV. This information could result in school enrollment behavior change to align with perceived expectations of study staff or because young women were motivated to prevent HIV. Second, compared to non-participants, trial participants were exposed to different networks likely to be supportive of school enrollment. Adult fieldworkers showed interest in the schooling of participants with yearly in-person data collection and monthly in-school data collection. Data were collected in 'camps' wherein trial participants were transported to study offices annually for a half-day of surveys and blood tests, and entertaining activities during wait periods (e.g. fingernail painting, photograph taking, magazine reading). This protocol could have fostered a cohesive group environment among trial participants resulting in positive peer pressure to maintain school enrollment. There is a growing body of evidence that interventions providing a safe space with adult mentorship and peer support can have positive outcomes for young women in sub-Saharan Africa,[24-26] a pathway that may have been activated with trial participation. Finally, trial participation provided access to certain health and social services that may have otherwise been inaccessible, including annual HIV and HSV-2 testing and counseling, linkage to care for those who tested positive, and linkage to social work services for young women who reported experiences of sexual abuse. These services may have enabled young

For peer review only

For peer review only - http://bmjopen.bmj.com/site/about/guidelines.xhtml

women who would have otherwise struggled with serious physical and mental health outcomes to remain in school.

Associations between trial participation and school enrollment were strongest in older age groups. The small differences observed in the youngest age group are understandable as they were under the age-limit of compulsory education with requirements to remain in school regardless of trial influence. For the oldest age group, trial selection criteria for lower grade levels meant they were older than expected for their grade, and suggested a history of grade repetition or temporary drop-out. That the trial protocol may have contributed to keeping older teens in school is significant as the transition to adulthood carries extremely high HIV risk.[23]

This study was fairly unusual in that data were available on key study outcomes for the underlying population from which study participants were drawn. The Agincourt HDSS routinely collects school enrollment data on all residents in the study area and we were able to leverage those data to assess differences between trial participants and non-participants. The majority of epidemiologic studies do not have the benefit of complete background data on the target population, and, as such, sample selection and Hawthorne effects are rarely empirically assessed.[5 10 12 14] However, when Hawthorne effects are assessed, the direction of the relationship between research participation and healthy outcomes tends to be positive, in line with our findings of improved school enrollment outcomes.

It is important to note that data on HIV incidence, the primary endpoint of HPTN 068, were not available for the underlying target population. We speculate that the improved schooling trajectories we observed in trial participants likely resulted in reduced risk of HIV acquisition.[17] Continued schooling is strongly associated with HIV prevention and reduced sexual risk outcomes in young women in sub-Saharan Africa[18 27 28] and we observed lower HIV incidence

For peer review only - http://bmjopen.bmj.com/site/about/guidelines.xhtml

(1.8%) than expected among trial participants (3%). However, we cannot say with certainty that the association between trial participation and school enrollment extended to HIV protection.

The potential for uncontrolled confounding requires that our results be interpreted cautiously. The differences we attribute to the Hawthorne effect were estimated in an observational dataset. Initial screens for trial eligibility were performed based on age data maintained by the Agincourt HDSS and 82% of the eligible young women approached went on to enroll in the study, a fairly high response rate.[17] Still, it is plausible that those who refused participation were different from those who consented in ways that are also related to future school enrollment trajectories. Although we controlled for key socio-demographic characteristics that we theorized could be related to both trial participation and school enrollment, the possibility for bias from unmeasured confounding remains.

We offer three key conclusions from this study. First, epidemiologists should give greater weight at the planning, analysis, and dissemination stages to identifying how sample selection and Hawthorne effects can be minimized, assessed, and discussed. Prioritizing research with well-defined target populations in areas with ongoing background data collection (e.g. HDSS centers) would improve researchers' abilities to empirically assess the external validity of their findings. Second, the sample selection effect we observed highlights how school-based samples can differ in important ways from non-school based samples in terms of underlying risk. Interventions focused on school-going adolescents may not reach those most in need of prevention, an anticipated issue that was ultimately difficult to avoid given HPTN 068 design. Third, the Hawthorne-specific findings suggest that aspects of the HPTN 068 protocol could potentially be adapted for school retention interventions to prevent HIV. If the relationship we observed is causal, the trial protocol increased school enrollment at a magnitude similar to targeted cash transfer interventions and other fairly resource-intensive school retention

For peer review only - http://bmjopen.bmj.com/site/about/guidelines.xhtml

interventions in sub-Saharan Africa,[21 29-31] despite the actual contact with the young women being limited to annual visits. Future work should examine key elements of the study protocol – adult mentorship, peer support, school attendance monitoring, messaging around the link between school and HIV, routine HIV/STI testing and linkage to care – to better understand their relationship with school retention and HIV acquisition.

For peer review only

For peer review only - http://bmjopen.bmj.com/site/about/guidelines.xhtml

Acknowledgments: The Agincourt Health and socio-Demographic Surveillance System is supported by the Wellcome Trust (058893/Z/99/A; 069683/Z/02/Z; 085477/Z/08/Z; 085477/B/08/Z), the University of the Witwatersrand, and Medical Research Council, South Africa. HPTN 068 was supported by Award Numbers UM1 AI068619 (HPTN Leadership and Operations Center), UM1AI068617 (HPTN Statistical and Data Management Center), and UM1AI068613 (HPTN Laboratory Center) from the National Institute of Allergy and Infectious Diseases, the National Institute of Mental Health and the National Institute on Drug Abuse of the National Institutes of Health. This work was also supported by NIMH (R01MH087118) and the Carolina Population Center and its NIH Center grant (P2C HD050924). The content is solely the responsibility of the authors and does not necessarily represent the official views of the National Institutes of Health.

For peer review only

For peer review only - http://bmjopen.bmj.com/site/about/guidelines.xhtml

Figure 1. Timeline of Agincourt HDSS education data collection and HPTN 068 trial duration

For peer review only

Table 1. Socio-demographic characteristics of a cohort of 3889 young women between the ages of 13-20 years in rural Agincourt, South Africa, by participation in the HPTN 068 trial

	Total (n=3889)		Trial participant[2] (n=1720)		Non-participant (n=2169)		
	N	%	N	%	N	%	p-value[1]
**Age in 2011**							<0.0001
11 - 12	188	4.8	26	1.5	162	7.5	
13 - 14	1270	32.7	637	37.0	633	29.2	
15 - 16	1591	40.9	793	46.1	798	36.8	
17 - 18	667	17.2	239	13.9	428	19.7	
19 - 20	173	4.5	25	1.5	148	6.8	
**SES***							0.003
At or above median	1778	50	853	52.7	925	47.8	
Below median	1778	50	766	47.3	1012	52.3	
Missing	333		101		232		
**Household size***							0.5
Mean	8.4		8.4		8.5		
SD	4.2		4.0		4.4		
Missing	46		14		32		
**Gender of HH head***							0.3
Female	1590	41.7	691	40.8	899	42.4	
Male	2224	58.3	1004	59.2	1220	57.6	
Missing	75		25		50		
**Household head educational attainment***							0.02
<Grade 12	3062	86.3	1398	87.8	1664	85.1	
Grad 12 or higher	486	13.7	194	12.2	292	14.9	
Missing	341		128		213		
**Country of origin***							0.2
South Africa	2315	59.6	1046	60.9	1269	58.6	
Mozambique	1570	40.4	673	39.2	897	41.4	
Missing	4		1		3		
**Childbearing before 2011**							<0.0001
Yes	274	7.1	67	3.9	207	9.5	
No	3615	93.0	1653	96.1	1962	90.5	
**Intervention arm**							
Control			820	50.4			
Intervention			806	49.6			
**2011 school enrollment**							<0.0001
Yes	3508	95.7	1637	99.2	1871	92.9	
No	158	4.3	14	0.9	144	7.2	
Missing	223		69		154		
**2015 school enrollment**							<0.0001
Yes	2465	74.2	1234	19	1231	68.5	
No	856	25.8	290	81	566	31.5	
Missing	568		196		372		

[1]p-values for categorical variables are from chi-square tests and for continuous variables are from t-tests

[2]Due to restrictions in the cohort construction to maintain comparable groups with respect to age and education status in 2009, not all of the 2533 HPTN 068 participants are represented.

*Measured in 2009

For peer review only - http://bmjopen.bmj.com/site/about/guidelines.xhtml

Table 2. The relationship between HPTN 068 trial participation and school enrollment in 2011 and 2015, stratified by age, in the full cohort and the cohort restricted to those enrolled in school in 2011

	Enrollment	N	Percent enrolled	RD (95% CI)	aRD[1] (95% CI)
2011: Full cohort					
**All ages**					
Trial participant	1637	1651	99.2 (98.7, 99.6)	6.3 (5.1, 7.5)	2.9 (-0.7, 6.5)
Non-trial participant	1871	2015	92.9 (91.7, 94.0)	1	1
**Ages 11-15**					
Trial participant	1036	1038	99.8 (99.5, 1.00)	0.6 (0.0, 1.2)	0.4 (-5.2, 5.9)
Non-trial participant	1115	1124	99.2 (98.6, 99.7	1	1
**Ages 16-17**					
Trial participant	532	539	98.7 (97.8, 99.7)	6.9 (4.6, 9.2)	6.3 (0.5, 12.2)
Non-trial participant	593	646	91.8 (89.7, 93.9)	1	1
**Ages 18-20**					
Trial participant	69	74	93.2 (87.5, 99.0)	26.7 (18.5, 34.9)	19.5 (5.6, 33.3)
Non-trial participant	163	245	66.5 (60.6, 72.4)	1	1
2015: Full cohort					
**All ages**					
Trial participant	1234	1524	81.0 (79.0, 83.0)	12.5 (9.6, 15.4)	6.8 (3.4, 10.2)
Non-trial participant	1231	1797	68.5 (66.4, 70.7)	1	1
**Ages 11-15**					
Trial participant	837	952	87.9 (85.9, 90.0)	3.8 (0.1, 6.9)	1.9 (-1.3, 5.0)
Non-trial participant	833	990	84.1 (81.9, 86.5)	1	1
**Ages 16-17**					
Trial participant	361	503	71.8 (67.9, 75.8)	12.5 (6.9, 18.1)	12.7 (6.7, 18.7)
Non-trial participant	346	584	59.3 (55.4, 63.4)	1	1
**Ages 18-20**					
Trial participant	36	69	52.2 (41.6, 65.4)	28.9 (15.8, 41.9)	29.7 (16.1, 43.3)
Non-trial participant	52	223	23.3 (18.4, 29.6)	1	1
2015: Restricted cohort[2]					
**All ages**					
Trial participant	1234	1510	81.7 (79.8, 83.7)	7.3 (4.5, 10.2)	4.9 (1.5, 8.3)
Non-trial participant	1231	1655	74.4 (72.3, 76.5)	1	1
**Ages 11-15**					
Trial participant	837	950	88.1 (86.1, 90.2)	3.2 (0.2, 6.3)	1.8 (-1.3, 5.0)
Non-trial participant	833	982	84.8 (82.6, 87.1)	1	1
**Ages 16-17**					
Trial participant	361	496	72.8 (69.0, 76.8)	7.6 (1.9, 13.3)	7.9 (1.9, 14.0)
Non-trial participant	346	531	65.2 (61.2, 69.3)	1	1
**Ages 18-20**					
Trial participant	36	64	56.3 (45.3, 69.8)	19.6 (5.1, 34.1)	22.8 (7.3, 38.2)
Non-trial participant	52	142	36.6 (29.5, 45.5)	1	1

[1]Adjusted for socio-economic status (coded dichotomously at median household asset index score), country of origin (South African versus Mozambican), educational attainment of household head (coded dichotomously at Grade 12 attainment), gender of household head (male versus female), household size (coded linearly), and pre-2011 childbearing (yes versus no). Models that are not age-stratified are also adjusted for age coded in two-year categories.

[2]Restricted to all young women who were enrolled in school in 2011

RD=risk difference; aRD=adjusted risk difference; CI=confidence interval

For peer review only - http://bmjopen.bmj.com/site/about/guidelines.xhtml

Table 3. Sensitivity analysis comparing differences in school enrollment between HPTN 068 trial participants in the control arm only and non-trial participants. School enrollment outcome was analyzed in 2011 and 2015 and the analysis was stratified by age, in the full cohort and the cohort restricted to those enrolled in school in 2011

	Enrollment	N	Percent enrolled	RD (95% CI)	aRD[1] (95% CI)
2011: Full cohort					
**All ages**					
Trial participant (control)	862	872	98.9 (98.2, 99.6)	6.0 (4.7, 7.3)	3.2 (-1.4, 7.7)
Non-trial participant	1871	2015	92.9 (91.7, 94.0)	1	1
**Ages 11-15**					
Trial participant (control)	543	544	99.8 (99.5, 1.00)	0.6 (0.0, 1.3)	0.4 (-7.3, 8.0)
Non-trial participant	1115	1124	99.2 (98.6, 99.7	1	1
**Ages 16-17**					
Trial participant (control)	280	285	98.3 (96.7, 99.8)	6.5 (3.8, 9.1)	5.7 (-0.9, 12.3)
Non-trial participant	593	646	91.8 (89.7, 93.9)	1	1
**Ages 18-20**					
Trial participant (control)	39	43	90.7 (82.0, 99.4)	24.2 (13.7, 34.7)	17.7 (2.6, 32.8)
Non-trial participant	163	245	66.5 (60.6, 72.4)	1	1
2015: Full cohort					
**All ages**					
Trial participant (control)	653	813	80.3 (77.6, 83.1)	11.8 (8.3, 15.3)	6.9 (3.6, 10.3)
Non-trial participant	1231	1797	68.5 (66.4, 70.7)	1	1
**Ages 11-15**					
Trial participant (control)	448	504	88.9 (86.2, 91.6)	4.8 (1.2, 8.3)	2.4 (-1.3, 6.1)
Non-trial participant	833	990	84.1 (81.9, 86.5)	1	1
**Ages 16-17**					
Trial participant (control)	186	269	69.1 (63.6, 74.7)	9.9 (3.1, 16.7)	9.7 (2.6, 16.8)
Non-trial participant	346	584	59.3 (55.4, 63.4)	1	1
**Ages 18-20**					
Trial participant (control)	19	40	47.5 (32.0, 63.0)	24.2 (7.7, 40.6)	21.5 (4.9, 38.1)
Non-trial participant	52	223	23.3 (18.4, 29.6)	1	1
2015: Restricted cohort[2]					
**All ages**					
Trial participant (control)	653	803	81.3 (78.6, 84.0)	6.9 (3.5, 10.4)	4.5 (1.3, 7.6)
Non-trial participant	1231	1655	74.4 (72.3, 76.5)	1	1
**Ages 11-15**					
Trial participant (control)	448	503	89.1 (86.3, 91.8)	4.2 (0.7, 7.8)	2.5 (-1.2, 6.2)
Non-trial participant	833	982	84.8 (82.6, 87.1)	1	1
**Ages 16-17**					
Trial participant (control)	186	264	70.5 (65.0, 76.0)	5.3 (-1.5, 12.1)	5.2 (-2.0, 12.3)
Non-trial participant	346	531	65.2 (61.2, 69.3)	1	1
**Ages 18-20**					
Trial participant (control)	19	36	52.8 (36.5, 69.1)	16.2 (-2.0, 34.3)	13.8 (-5.7, 33.4)
Non-trial participant	52	142	36.6 (29.5, 45.5)	1	1

[1]Adjusted for socio-economic status (coded dichotomously at median household asset index score), country of origin (South African versus Mozambican), educational attainment of household head (coded dichotomously at Grade 12 attainment), gender of household head (male versus female), household size (coded linearly), and pre-2011 childbearing (yes versus no). Models that are not age-stratified are also adjusted for age coded in two-year categories.

[2]Restricted to all young women who were enrolled in school in 2011

RD=risk difference; aRD=adjusted risk difference; CI=confidence interval

For peer review only - http://bmjopen.bmj.com/site/about/guidelines.xhtml

For peer review only

For peer review only - http://bmjopen.bmj.com/site/about/guidelines.xhtml

References

1. Victora CG, Habicht J-P, Bryce J. Evidence-based public health: moving beyond randomized trials. *American journal of public health* 2004;94(3):400-05.
2. Rychetnik L, Frommer M, Hawe P, et al. Criteria for evaluating evidence on public health interventions. *Journal of Epidemiology & Community Health* 2002;56(2):119-27.
3. Westreich D, Edwards JK, Rogawski ET, et al. Causal impact: epidemiological approaches for a public health of consequence. *American journal of public health* 2016;106(6):1011.
4. Adair JG. The Hawthorne effect: A reconsideration of the methodological artifact. *Journal of applied psychology* 1984;69(2):334.
5. McCarney R, Warner J, Iliffe S, et al. The Hawthorne Effect: a randomised, controlled trial. *BMC medical research methodology* 2007;7(1):1.
6. Steckler A, McLeroy KR. The importance of external validity: American Public Health Association, 2008.
7. Cole SR, Stuart EA. Generalizing evidence from randomized clinical trials to target populations the actg 320 trial. *American journal of epidemiology* 2010;172(1):107-15.
8. Hernán MA, VanderWeele TJ. Compound treatments and transportability of causal inference. *Epidemiology (Cambridge, Mass)* 2011;22(3):368.
9. Westreich D, Edwards JK. Invited commentary: every good randomization deserves observation. *American journal of epidemiology* 2015;182(10):857-60.
10. Braunholtz DA, Edwards SJ, Lilford RJ. Are randomized clinical trials good for us (in the short term)? Evidence for a "trial effect". *Journal of clinical epidemiology* 2001;54(3):217-24.
11. Robinson E, Hardman CA, Halford JC, et al. Eating under observation: a systematic review and meta-analysis of the effect that heightened awareness of observation has on laboratory measured energy intake. *The American journal of clinical nutrition* 2015 doi: 10.3945/ajcn.115.111195 [published Online First: 2015/07/17]
12. Menezes P, Miller WC, Wohl DA, et al. Does HAART efficacy translate to effectiveness? Evidence for a trial effect. *PloS one* 2011;6(7):e21824.
13. McCambridge J, Kypri K, Elbourne D. In randomization we trust? There are overlooked problems in experimenting with people in behavioral intervention trials. *J Clin Epidemiol* 2014;67(3):247-53. doi: 10.1016/j.jclinepi.2013.09.004 [published Online First: 2013/12/10]
14. McCambridge J, Witton J, Elbourne DR. Systematic review of the Hawthorne effect: new concepts are needed to study research participation effects. *J Clin Epidemiol* 2014;67(3):267-77. doi: 10.1016/j.jclinepi.2013.08.015 [published Online First: 2013/11/28]
15. Padian NS, McLOY SI, Balkus JE, et al. Weighing the gold in the gold standard: challenges in HIV prevention research. *AIDS (London, England)* 2010;24(5):621.
16. Pettifor A, MacPhail C, Selin A, et al. HPTN 068: a randomized control trial of a conditional cash transfer to reduce HIV infection in young women in South Africa—study design and baseline results. *AIDS and behavior* 2016;20(9):1863-82.
17. Pettifor A, MacPhail C, Hughes JP, et al. The effect of a conditional cash transfer on HIV incidence in young women in rural South Africa (HPTN 068): a phase 3, randomised controlled trial. *The Lancet Global Health* 2016;4(12):e978-e88.
18. Rosenberg M, Pettifor A, Miller WC, et al. Relationship between school dropout and teen pregnancy among rural South African young women. *International journal of epidemiology* 2015:dyv007.

For peer review only

19. Strassburg S, Meny-Gibert S, Russell B. Left unfinished: Temporary absence and drop-out from South African schools. Access to Education. Johannesburg, South Africa: Social Surveys Africa, 2010.
20. Department of Basic Education Republic of South Africa. Education for All 2014 Progress Report: South Africa. Pretoria, 2014.
21. Baird S, Ferreira FH, Özler B, et al. Relative effectiveness of conditional and unconditional cash transfers for schooling outcomes in developing countries: a systematic review. *Campbell systematic reviews* 2013;9(8)
22. Kahn K, Collinson MA, Gomez-Olive FX, et al. Profile: Agincourt Health and Socio-demographic Surveillance System. *International journal of epidemiology* 2012;41(4):988-1001. doi: 10.1093/ije/dys115 [published Online First: 2012/08/31]
23. Gómez-Olivé FX, Angotti N, Houle B, et al. Prevalence of HIV among those 15 and older in rural South Africa. *AIDS care* 2013;25(9):1122-28.
24. Erulkar AS, Muthengi E. Evaluation of Berhane Hewan: a program to delay child marriage in rural Ethiopia. *International Perspectives on Sexual and Reproductive Health* 2009:6-14.
25. Bandiera O, Buehren N, Burgess R, et al. Empowering adolescent girls: Evidence from a randomized control trial in Uganda. *Washington, DC: World Bank* 2012
26. Austrian K, Hewett P, Soler-Hampejsek E, et al. Adolescent Girls Empowerment Programme: Research and Evaluation Mid-Term Technical Report. Lusaka, Zambia: Population Council, 2016.
27. Pettifor AE, Levandowski BA, MacPhail C, et al. Keep them in school: the importance of education as a protective factor against HIV infection among young South African women. *International journal of epidemiology* 2008;37(6):1266-73. doi: 10.1093/ije/dyn131 [published Online First: 2008/07/11]
28. Jukes M, Simmons S, Bundy D. Education and vulnerability: the role of schools in protecting young women and girls from HIV in southern Africa. *AIDS (London, England)* 2008;22 Suppl 4:S41-56. doi: 10.1097/01.aids.0000341776.71253.04 [published Online First: 2008/12/17]
29. Cho H, Hallfors DD, Mbai, II, et al. Keeping adolescent orphans in school to prevent human immunodeficiency virus infection: evidence from a randomized controlled trial in Kenya. *The Journal of adolescent health : official publication of the Society for Adolescent Medicine* 2011;48(5):523-6. doi: 10.1016/j.jadohealth.2010.08.007 [published Online First: 2011/04/20]
30. Hallfors D, Cho H, Rusakaniko S, et al. Supporting adolescent orphan girls to stay in school as HIV risk prevention: evidence from a randomized controlled trial in Zimbabwe. *Am J Public Health* 2011;101(6):1082-8. doi: AJPH.2010.300042 [pii]
10.2105/AJPH.2010.300042 [published Online First: 2011/04/16]
31. Baird S, Chirwa E, McIntosh C, et al. The short-term impacts of a schooling conditional cash transfer program on the sexual behavior of young women. *Health economics* 2010;19 Suppl:55-68. doi: 10.1002/hec.1569 [published Online First: 2009/12/01]

For peer review only

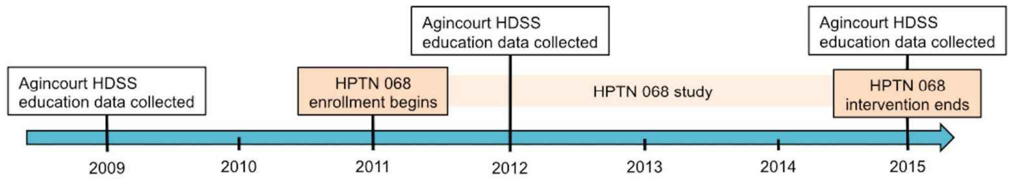

Figure 1. Timeline of Agincourt HDSS education data collection and HPTN 068 trial duration

381x285mm (300 x 300 DPI)

For peer review only - http://bmjopen.bmj.com/site/about/guidelines.xhtml

STROBE Statement—Checklist of items that should be included in reports of ***cohort studies***

	Item No	Recommendation	Page number
**Title and abstract**	1	(*a*) Indicate the study's design with a commonly used term in the title or the abstract	2
		(*b*) Provide in the abstract an informative and balanced summary of what was done and what was found	2
**Introduction**			
Background/rationale	2	Explain the scientific background and rationale for the investigation being reported	4
Objectives	3	State specific objectives, including any prespecified hypotheses	5
**Methods**			
Study design	4	Present key elements of study design early in the paper	5-7
Setting	5	Describe the setting, locations, and relevant dates, including periods of recruitment, exposure, follow-up, and data collection	5-7
Participants	6	(*a*) Give the eligibility criteria, and the sources and methods of selection of participants. Describe methods of follow-up	5-7
		(*b*) For matched studies, give matching criteria and number of exposed and unexposed	
Variables	7	Clearly define all outcomes, exposures, predictors, potential confounders, and effect modifiers. Give diagnostic criteria, if applicable	7
Data sources/ measurement	8*	For each variable of interest, give sources of data and details of methods of assessment (measurement). Describe comparability of assessment methods if there is more than one group	*7*
Bias	9	Describe any efforts to address potential sources of bias	8
Study size	10	Explain how the study size was arrived at	6-7
Quantitative variables	11	Explain how quantitative variables were handled in the analyses. If applicable, describe which groupings were chosen and why	7-8
Statistical methods	12	(*a*) Describe all statistical methods, including those used to control for confounding	8
		(*b*) Describe any methods used to examine subgroups and interactions	8
		(*c*) Explain how missing data were addressed	Table 1
		(*d*) If applicable, explain how loss to follow-up was addressed	Table 1
		(*e*) Describe any sensitivity analyses	n/a
**Results**			
Participants	13*	(a) Report numbers of individuals at each stage of study—eg numbers potentially eligible, examined for eligibility, confirmed eligible, included in the study, completing follow-up, and analysed	9
		(b) Give reasons for non-participation at each stage	n/a
		(c) Consider use of a flow diagram	n/a
Descriptive data	14*	(a) Give characteristics of study participants (eg demographic, clinical, social) and information on exposures and potential confounders	9
		(b) Indicate number of participants with missing data for each variable of interest	Table 1
		(c) Summarise follow-up time (eg, average and total amount)	n/a
Outcome data	15*	Report numbers of outcome events or summary measures over time	Table 1
Main results	16	(*a*) Give unadjusted estimates and, if applicable, confounder-adjusted estimates and their precision (eg, 95% confidence interval). Make clear	Table 2

For peer review only

For peer review only - http://bmjopen.bmj.com/site/about/guidelines.xhtml

		which confounders were adjusted for and why they were included	
		(*b*) Report category boundaries when continuous variables were categorized	Table 2
		(*c*) If relevant, consider translating estimates of relative risk into absolute risk for a meaningful time period	Table 2
Other analyses	17	Report other analyses done—eg analyses of subgroups and interactions, and sensitivity analyses	Table 2
**Discussion**			
Key results	18	Summarise key results with reference to study objectives	10
Limitations	19	Discuss limitations of the study, taking into account sources of potential bias or imprecision. Discuss both direction and magnitude of any potential bias	10-13
Interpretation	20	Give a cautious overall interpretation of results considering objectives, limitations, multiplicity of analyses, results from similar studies, and other relevant evidence	10-13
Generalisability	21	Discuss the generalisability (external validity) of the study results	10-13
**Other information**			
Funding	22	Give the source of funding and the role of the funders for the present study and, if applicable, for the original study on which the present article is based	14

*Give information separately for exposed and unexposed groups.

Note: An Explanation and Elaboration article discusses each checklist item and gives methodological background and published examples of transparent reporting. The STROBE checklist is best used in conjunction with this article (freely available on the Web sites of PLoS Medicine at http://www.plosmedicine.org/, Annals of Internal Medicine at http://www.annals.org/, and Epidemiology at http://www.epidem.com/). Information on the STROBE Initiative is available at http://www.strobe-statement.org.

For peer review only

For peer review only - http://bmjopen.bmj.com/site/about/guidelines.xhtml
